# Directing Uphill Strand Displacement with an Engineered Superhelicase

**DOI:** 10.1021/acssynbio.3c00452

**Published:** 2023-10-16

**Authors:** Helena Hall-Thomsen, Shavier Small, Momcilo Gavrilov, Taekjip Ha, Rebecca Schulman, Pepijn Gerben Moerman

**Affiliations:** † Chemical & Biomolecular Engineering, Johns Hopkins University, Baltimore, Maryland 21218, United States; ‡ Biophysics and Biophysical Chemistry, Johns Hopkins University, Baltimore, Maryland 21218, United States; § Biomedical Engineering, Johns Hopkins University, Baltimore, Maryland 21218, United States; ∥ Howard Hughes Medical Institute, Chevy Chase, Maryland 20815, United States; ⊥ Computer Science, Johns Hopkins University, Baltimore, Maryland 21218, United States; # Chemistry, Johns Hopkins University, Baltimore, Maryland 21218, United States; ¶ Chemical Engineering and Chemistry, Eindhoven University of Technology, Eindhoven 5612 AP, Netherlands

**Keywords:** DNA nanotechnology, strand displacement reaction, helicase

## Abstract

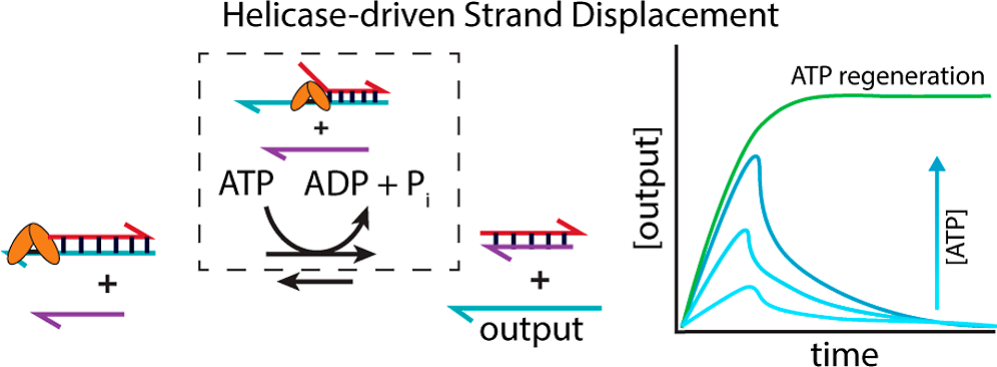

The ability to finely tune reaction rates and binding energies between components has made DNA strand displacement circuits promising candidates to replicate the complex regulatory functions of biological reaction networks. However, these circuits often lack crucial properties, such as signal turnover and the ability to transiently respond to successive input signals that require the continuous input of chemical energy. Here, we introduce a method for providing such energy to strand displacement networks in a controlled fashion: an engineered DNA helicase, Rep-X, that transiently dehybridizes specific DNA complexes, enabling the strands in the complex to participate in downstream hybridization or strand displacement reactions. We demonstrate how this process can direct the formation of specific metastable structures by design and that this dehybridization process can be controlled by DNA strand displacement reactions that effectively protect and deprotect a double-stranded complex from unwinding by Rep-X. These findings can guide the design of active DNA strand displacement regulatory networks, in which sustained dynamical behavior is fueled by helicase-regulated unwinding.

## Introduction

In living matter, chemically fueled reaction networks consume energy to regulate crucial biological functions such as metabolism,^1^ mitosis,^2^ and muscle contraction.^3^ Emulating the nonequilibrium reaction networks found in nature could provide routes to achieving homeostasis, oscillatory behavior, or chemical communication within synthetic, engineered chemical systems.^4−7^

DNA strands are ideal for studying reaction network design because both the thermodynamics and kinetics of their interactions can be finely tuned.^8^ For the former, due to the specificity of Watson–Crick base pairing, the degree of sequence complementarity between any two strands of a set determines their mutual binding strengths.^9^ For the latter, in displacement reactions where two DNA strands compete for binding to the same domain on a third strand, the lengths of the toeholds––single-stranded regions that initiate the reactions––control the rate constant across multiple orders of magnitude, and hence fine-tune the reaction rates,^10,11^ Because of this ability to control the thermodynamics and kinetics of reactions between DNA species, toehold-mediated strand displacement is the foundation for many DNA reaction networks, that can, among others, do calculations or recognize concentration patterns,^12,13^

**Figure 1:**
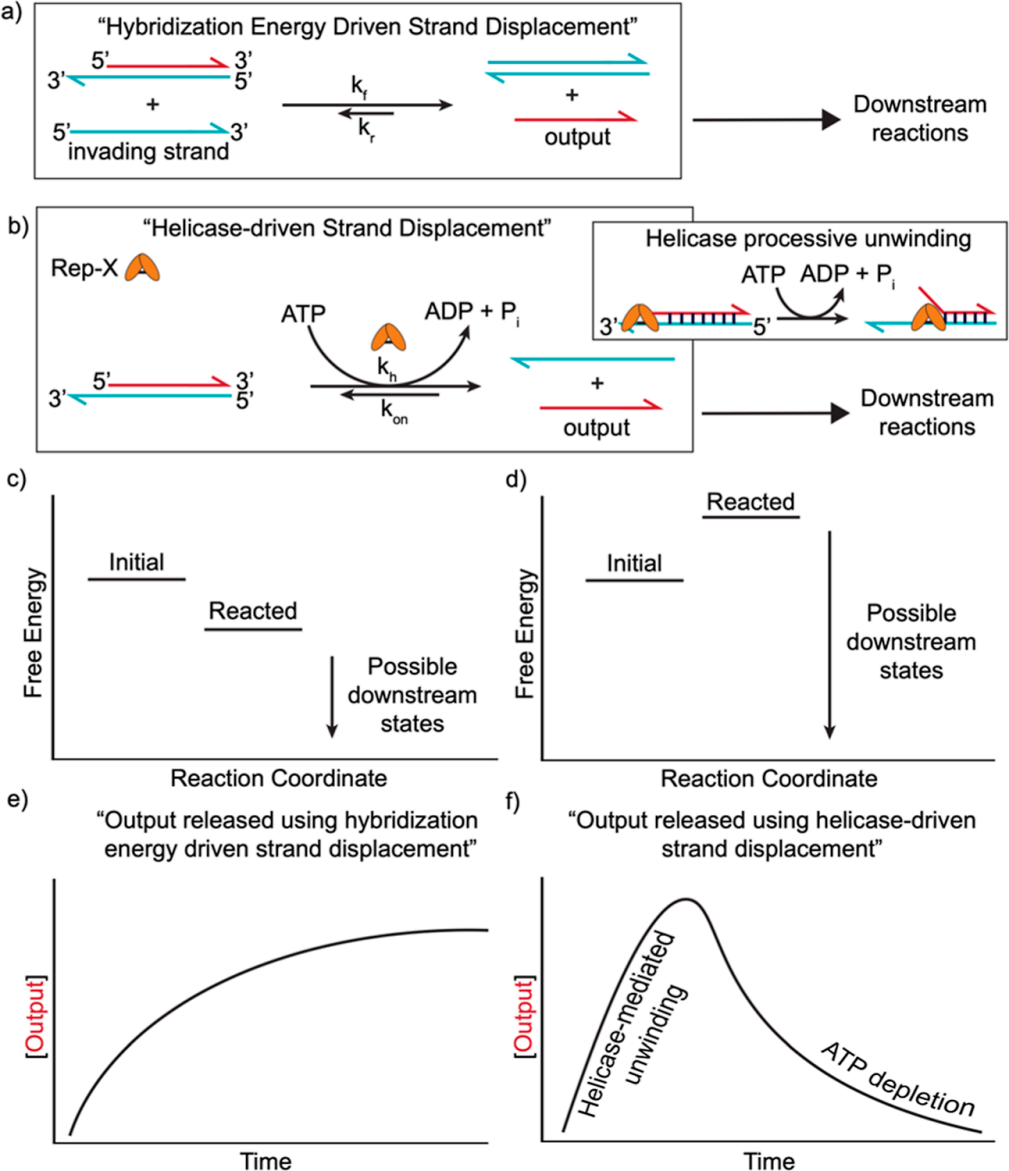
Comparison between hybridization energy-driven strand displacement and helicase-driven strand displacement. (a) Hybridization energy-driven strand displacement. In a typical toehold-mediated strand displacement reaction,^15^ the invading strand binds to the toehold of a partially double-stranded complex and displaces the red output strand, which can participate in downstream reactions. (b) Helicase-driven strand displacement. Helicase binds to a target complex containing the output and unwinds the strands, allowing the output to participate in downstream reactions. Helicase-mediated processive unwinding, shown in the inset, is initiated by the binding of helicase to a 3′ overhang. (c) Free-energy diagram of hybridization energy-driven strand displacement. The displacement of the output strand by the invading strand lowers the free energy of the system because the products of the reaction have more hybridized nucleotides than the reactants. (d) Free-energy diagram of helicase-driven strand displacement. The helicase-mediated unwinding of the input complex into two separate strands increases the free energy of the DNA strands in the system by consuming energy from ATP. (e) Output release over time through hybridization energy-driven displacement. The concentration of the free output is expected to monotonically increase and plateau as the system moves to the thermodynamically favored reaction coordinate. (f) Output release over time through helicase-driven strand displacement. The concentration of free output is expected to increase initially as the helicase unwinds the input complex. Then, after ATP is depleted, the unwinding rate drops, causing the concentration of single-stranded outputs to drop as the strands rehybridize and revert to the complexed state.

Biological reaction networks can sustain their responsive behavior and respond to multiple inputs because they consume fuel to continually maintain a far-from-equilibrium state and, in the absence of input, return to a specific initial state. Strand displacement circuits rely on fuel only from metastable DNA complexes; the energy stored in these complexes is limited, as compared to the energy stored in chemical bonds, and regulating its use is challenging because side or leak reactions cause spontaneous fuel consumption^14,15^ (Figure 1a,c). Dissipative DNA nanotechnology aims to devise methods to switch DNA complexes between reactive and unreactive states by connecting them to exergonic chemical reactions.^16^ Such approaches could make it possible to achieve more precise and sustained control of driven chemical systems than approaches that harness only the energy of metastable DNA complexes.

A variety of dissipative approaches have been explored to create DNA circuits that exhibit repeated transient responses. RNA can be a transient signal that is produced by RNA polymerase^17^ and degraded by RNase H,^18,19^ ligases and restriction enzymes have degraded or altered DNA,^20^ and chemical methylation of modified thymines has temporarily made hybridization more favorable.^21^ However, these methods rely on the degradation or modification of the nucleic acid strands themselves,^21−25^ rather than solely on the consumption of an external fuel, which limits their use in larger DNA strand displacement networks. Here, we introduce a different approach: helicase dehybridizes a double-stranded region of a DNA complex. This process “activates” a DNA strand that would otherwise be inactive, i.e., hybridized to its complement. The availability of active strands creates a driving force for downstream reactions without altering––either through degradation or chemical modification––any of the DNA strands involved.

The application of helicases to unwind double-stranded DNA (dsDNA) and make the constituent strands available for subsequent hybridization reactions exploits helicase’s innate ability to separate dsDNA to create single-stranded domains, which act as active sites for DNA duplication or repair.^26^ We base our tool for DNA nanotechnology on a common and well-studied helicase: Rep, a helicase primarily responsible for restarting stalled DNA replication.^27^ We use a modified version of Rep, the superhelicase Rep-X,^28^ which contains a non-native sulfur bridge that constraints it to the active closed conformation, improving its processivity and unwinding rate to develop dissipative DNA reaction processes. Like native Rep, Rep-X can only bind to single-stranded 3′ overhangs to start the unwinding of duplexes (Figure 1b). Therefore, the function and selectivity of Rep-X allows one to design systems in which Rep-X will unwind only specific complexes in each reaction network.^29^

In this work, we show how Rep-X’s strand-unwinding propensity can be leveraged to trigger transient and energetically uphill DNA strand displacement reactions by releasing the participating strands from a passive hybridized complex. This release, or unwinding of dsDNA, provides the opportunity for the single-stranded components to react with other strands before returning to their original complexed state residing at a thermodynamic minimum. We explore this class of dissipative (de)hybridization reactions using progressively more complex networks. First, we confirm that Rep-X can transiently release strands from a double-stranded complex and quantify the fraction of unhybridized DNA produced by Rep-X’s unwinding activity. Then, we show that the released strands can participate in downstream hybridization reactions to form transient metastable structures whose lifetimes are determined by Rep-X’s activity. Finally, we demonstrate how Rep-X might orchestrate signal amplification and molecular logic possibilities by developing a “lock–key”-style DNA circuit that when locked protects the double-stranded complex from hybridization and unwinding by Rep-X and can be unlocked using a DNA strand key. Overall, this work establishes design methods to use helicases in dissipative DNA reaction networks that could replicate some of the functions achieved in natural dissipative processes.

## Results and Discussion

### Rep-X Unwinds Double-Stranded DNA

**Figure 2:**
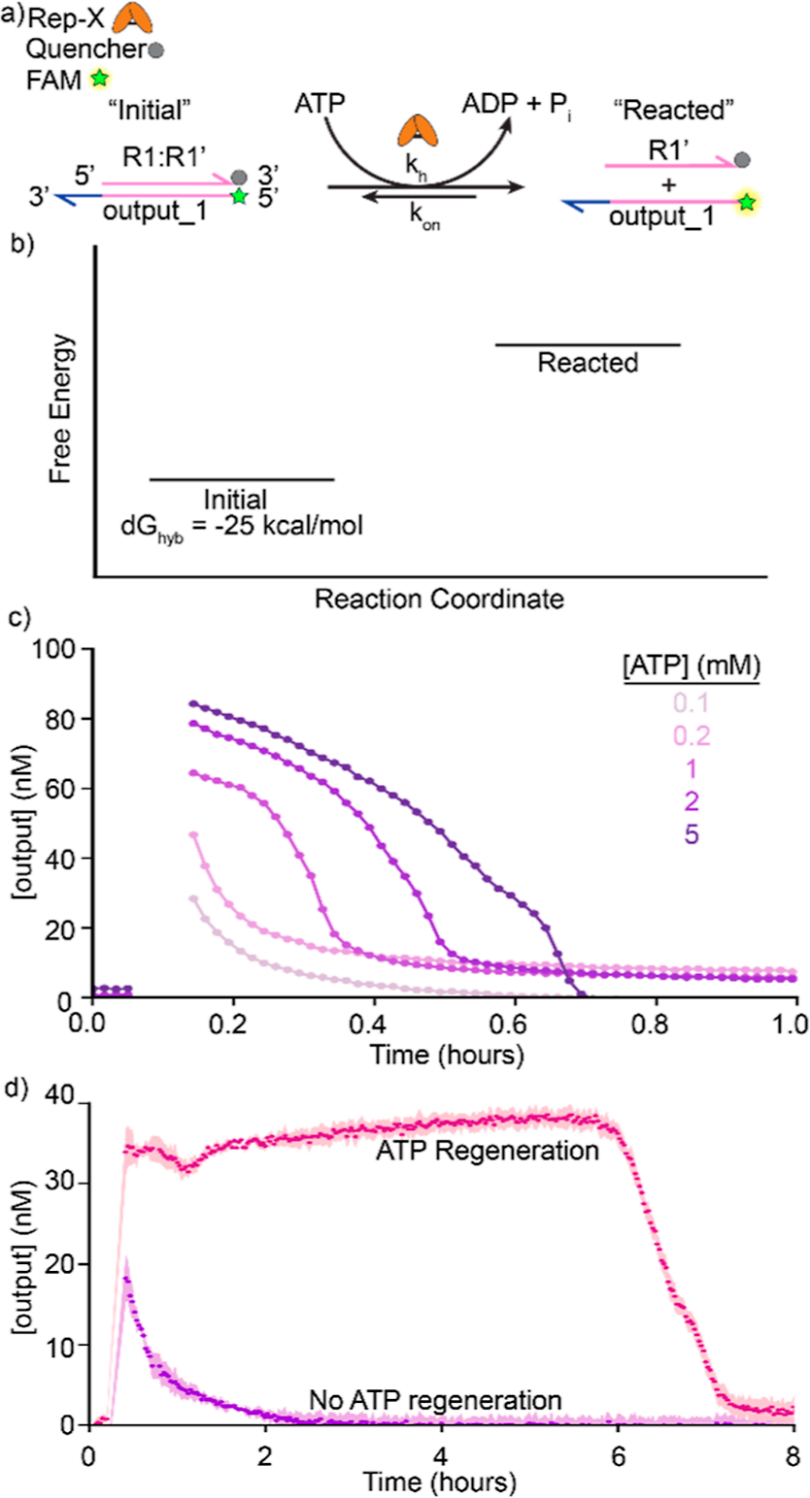
Two-strand helicase-mediated strand displacement. (a) Rep-X helicase unwinds the double-stranded R1:R1′ complex in a dissipative strand displacement reaction. A FAM fluorophore is attached to the 5′ end of output_1, whose sequence is the domain R1. An Iowa Black quencher is attached to the 3′ end of R1’. When unwound by Rep-X, the FAM fluorescence increases. (b) Free-energy diagram of helicase-mediated DNA dehybridization reaction from (a). The energetic cost of unwinding the R1:R1′ complex into two separate strands is the free energy of hybridization: −25 kcal/mol. Rep-X can unwind R1:R1′ to separate the complex into its higher energy states. The free energies were calculated in NUPACK using a reference concentration of 55.5 M (water in water) since we use an aqueous solution at low concentrations of solute. (c) Concentration of released output in the presence of Rep-X helicase and different concentrations of ATP. Samples contained 100 nM Rep-X, 100 nM R1:R1′, and either 0.1, 0.2, 1, 2, or 5 mM ATP. We observed a higher proportion of unwound complexes with increasing fuel (ATP) concentration. (d) Concentration of output as a consequence of R1:R1′ dehybridization by Rep-X helicase with ATP regeneration. Each sample contains 100 nM Rep-X, 50 nM R1:R1′, and 1 mM ATP; only the first sample contains an ATP regeneration system. In the sample with the ATP regeneration system (pink), the fraction of strands that are unwound (∼75%) is larger than that in the sample without ATP regeneration (∼35%) (purple). Additionally, the ATP regeneration system facilitates the sustaining of an unwound concentration for more than 5 h. In contrast, the concentration of unwound strand decreases immediately after its peak (∼25 min) without the ATP regeneration system. Shaded regions behind the data represent the standard deviations of triplicate experiments.

To evaluate Rep-X’s potential to facilitate dissipative strand displacement, we initially measured its capacity to unwind a double-stranded DNA complex with a 3′ overhang (i.e., a toehold). We used a two-stranded DNA reporter complex consisting of (a) a strand whose sequence comprises a 20-nucleotide binding domain, R1′, and (b) a strand (output_1) whose sequence comprises the complementary binding domain, R1, and a 5-nucleotide toehold on the 3′ end (Figure 2a and Table S1). This 5-nucleotide toehold on the 3′ end serves as a binding site for Rep-X, from which Rep-X can initiate the unwinding of the double-stranded R1:R1′ domain. To measure what fraction of this DNA complex is dehybridized by Rep-X, the 5′ end of output_1 was labeled with a fluorophore, while the 3′ end of the complementary strand was labeled with a quencher, an organic molecule that absorbs light in a range of wavelengths. When the strands are hybridized, the quencher absorbs the fluorescent signal from the output_1 strand, decreasing the fluorescence of the sample. Thus, the observed fluorescence is a measure of the fraction of unhybridized DNA complex due to Rep-X activity.

We found that the addition of Rep-X and adenosine triphosphate (ATP; Rep-X’s fuel) to a sample containing reporter complexes resulted in a fluorescence increase (Figure 2c), indicating that Rep-X unwinds the two strands by simultaneously converting the high-energy ATP into its lower energy waste product adenine diphosphate (ADP). Rep-X thus can move the DNA system from a lower free-energy state (hybridized) to an excited state (dehybridized) by harnessing energy from ATP, and this transition can be tracked through changes in fluorescence (Figure 2a,b). In our first set of experiments using 0.1, 0.2, 1, 2, and 5 mM ATP as fuel along with 100 nM Rep-X and 100 nM of R1:R1′, we observed increases in the concentration of free output_1 following the addition of Rep-X and ATP to the samples, indicating the successful separation of R1:R1′ in each case (Figure 2c). The greater the amount of ATP in the sample, the greater the proportion of R1:R1′ that Rep-X unwound. A maximum of around 80% unwound was observed in the sample containing 5 mM ATP. The ATP concentration also determined the relaxation rate of the system back to its lower free-energy state. Providing larger amounts of fuel allows Rep-X to unwind strands for a longer time, with the greatest relaxation time being around 40 min using 5 mM ATP. These results indicate that Rep-X can successfully unwind a simple dsDNA complex, and by adding more ATP, a higher proportion of complexes can be unwound over a longer time frame.

### ATP Regeneration Extends Rep-X’s Activity

The decrease over time in the fraction of single-stranded DNA (Figure 2c) demonstrates that Rep-X’s activity decays as ATP is depleted. The concentration of ATP also determines the fraction of active output_1 and the duration of time when active output_1 is present. Achieving a stable steady-state concentration of single-stranded output_1, which would be important for applications requiring a consistent response, such as DNA circuits designed to respond to inputs from a specific baseline state, might be achieved by incrementally adding ATP during the reaction, but that is impractical. Alternatively, we tested whether a steady-state unwinding rate could be achieved by keeping the ATP concentration constant using a second enzymatic reaction that regenerates ATP. This regenerative process is catalyzed by creatine phosphokinase (CPK), which transfers the phosphate from PCr (phosphocreatine) to ADP to form creatine and ATP.^30^ We found that the introduction of this ATP regeneration machinery enabled the fraction of output_1 in the unhybridized state to remain steady for more than 6 h. Additionally, the steady-state fraction of unhybridized output_1 was higher than that in the experiments we performed without the ATP regeneration reaction. Rep-X unwound around 75% of complexes using 1 mM ATP and the ATP regeneration machinery, but only 35% with 1 mM ATP alone (Figure 2d). Overall, adding the ATP regeneration machinery to the system in which Rep-X unwinds a DNA complex (Figure 2a) increases both the proportion of sample complexes unwound and the time at which there is a steady concentration of free output or output_1.

### Strands Released by Rep-X Can Participate in Downstream Reactions

**Figure 3:**
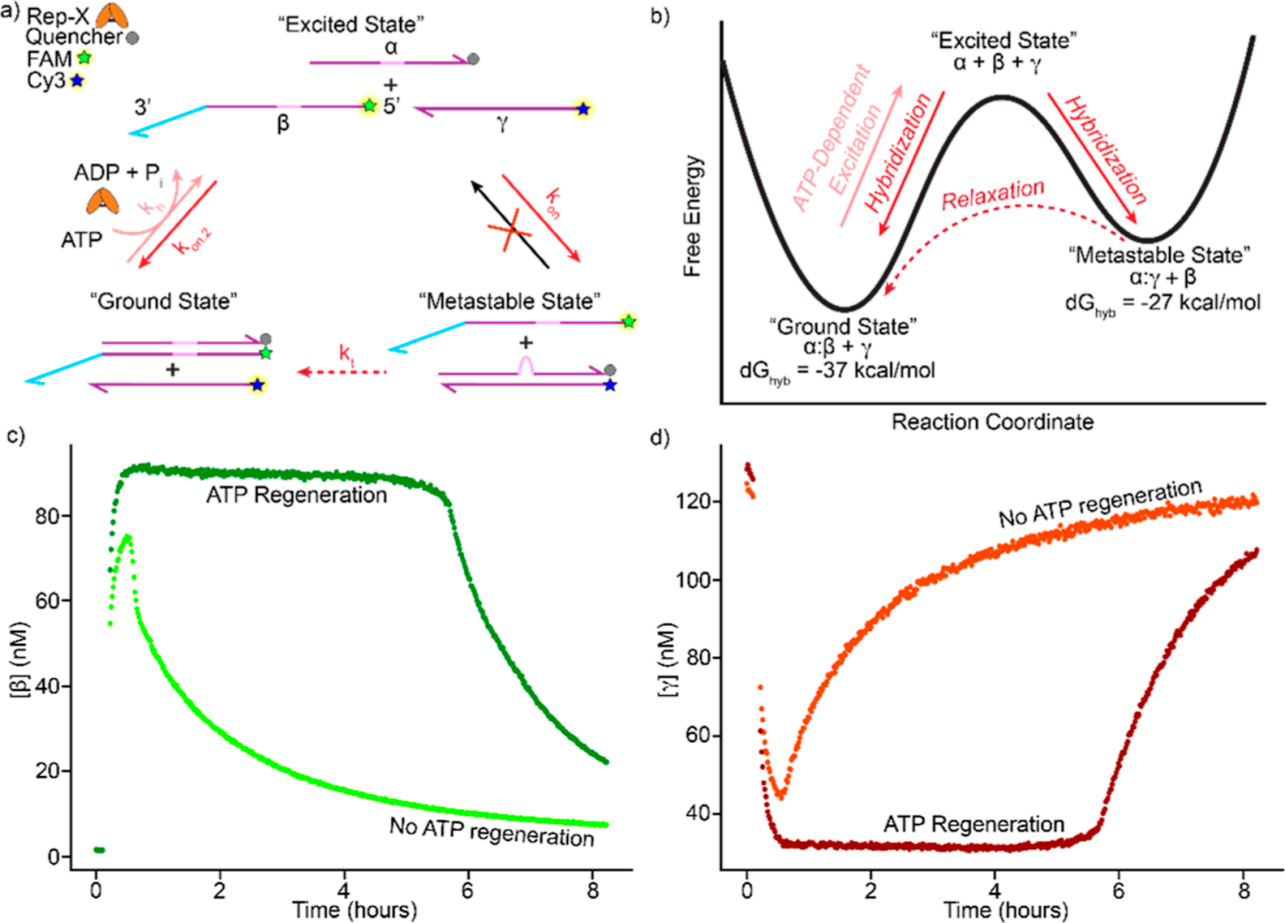
Helicase-mediated strand displacement can drive a downstream reaction. (a) Helicase-fueled dissipative strand displacement cycle coupled to a downstream reaction. There is a 10-nucleotide toehold on β in the α:β complex. Rep-X-mediated unwinding of the ground-state hybridized strands (α:β) results in an excited state where α, β, and γ are single-stranded. From the excited state, either α and β can hybridize to form an α/β complex to enter the ground state or α and γ can hybridize to enter the metastable state; the complex α/γ is transient. We tracked the states of this reaction using fluorescence measurements: α has an Iowa black quencher on its 3′ end, β has a 5-FAM fluorophore on its 5′ end, and γ has a 5-Cy3 fluorophore on its 5′ end. (b) Free-energy diagram of the α, β, and γ three-strand system. α:β and γ comprise the ground state (−37 kcal/mol); all single strands in solution comprise the excited state (0 kcal/mol); and α:γ and β comprise the metastable state (−27 kcal/mol). The values refer to the free energies of the hybridization process, starting in the “excited state” where all DNA molecules are single-stranded. The free energies were calculated with NUPACK using a reference concentration of 55.6 M (the concentration of water molecules in water), which is appropriate for an aqueous solution at low solute concentrations. When in the excited state, the system can either move back to the ground state or into the metastable state. The metastable state relaxes back to the equilibrium state over time. (c) Concentrations of β strands unwound by Rep-X with either ATP alone or ATP with regeneration machinery. With 100 nM Rep-X and 100 nM α, β, and γ, either 1 mM ATP (neon green) or 1 mM ATP with ATP regeneration machinery (dark green) was added to the solution. We observed a decline in Rep-X activity immediately after the maximum fraction of β was unwound in the sample containing only ATP. A steady fraction of unwound α/β was maintained for around 5 h in the sample containing ATP regeneration machinery. Both samples returned to or approached their baseline fluorescence after 8 h. (d) Concentrations of γ strands after Rep-X unwinds α:β using either ATP or ATP with regeneration machinery. With 100 nM Rep-X and 100 nM each of α, β, and γ, either 1 mM ATP (orange) or 1 mM ATP and regeneration machinery (dark red) was added to the solution. ∼65 and ∼75% of available γ strands hybridized with free α in the samples containing ATP and ATP with regeneration machinery, respectively. ∼75% of γ strands remained hybridized for about 5 h with ATP with regeneration machinery, whereas with ATP alone, the amount of free γ increased after reaching its minimum and returned to baseline after about 8 h.

Next, we set out to determine whether activated strands (i.e., strands exposed by unwinding of the complex by Rep-X) could participate in downstream reactions. We designed a three-strand system where one strand, alpha (α), is initially part of a double-stranded complex, alpha:beta (α:β), but could alternatively hybridize with another strand, γ (Figure 3a). In this system, the α:β complex with γ free in solution is the lower energy, ground state (−37 kcal/mol) because α and β have longer complementary regions than α and γ (23 vs 20 nucleotides). Rep-X can bind to the 10-nucleotide 3′ overhang on the β strand, unwind the α:β complex, and facilitate the system’s transition to an excited state (0 kcal/mol), where α, β, and γ are all unhybridized. From this state, an α strand has an equal probability of hybridizing with γ and entering the metastable state (−27 kcal/mol) or hybridizing with β and returning to the lowest energy state. The α:γ complexes are transient: α can rehybridize with β when α and γ become separated. By tracking the relative abundances of three states––ground, excited, and metastable––we sought to determine whether strands unwound by Rep-X can react with other DNA strands.

To track the binding states of α, β, and γ, we labeled the strands with fluorophores and quenchers (Figure 3a). The β-strand has a FAM fluorophore attached to its 5′ end with 5-nucleotide toehold for Rep-X to bind, γ has a Cy3 fluorophore attached to its 5′ end, and α has a quencher attached to its 3′ end. Hence, when γ is unhybridized, the observable fluorescence is primarily Cy3. When Rep-X unwinds α/β, the system’s transition into the metastable state should produce a decrease in Cy3 fluorescence and an increase in FAM fluorescence. We designed the system such that the α:β complex, but not the α:γ complex, has a single-stranded 3′ overhang and is susceptible to unwinding by Rep-X, so that Rep-X can control the system’s movement between energy states (Figure 3b).

We observed that Rep-X unwound the α:β complex, causing a ∼75% increase in the free β concentration and the corresponding decrease in the free γ concentration by ∼65% (Figure 3c,d). Thus, Rep-X catalyzed a transition from the ground state to the metastable state. After around 8 h, the system relaxed back to its ground state, as indicated by a decrease in the concentration of β and an increase in the concentration of γ back to its initial value. Notably, the free γ concentration reaches a minimum approximately 1 min after the concentration of free β concentration peaks. This means that the hybridization of α and γ is fast compared to the unwinding of α/β and that the lifetime of the excited, unhybridized state is short. Rep-X unwinds a similar proportion of α:β complexes as it did with the strands in the two-strand system, but the three-strand system takes longer to return to the initial state after ATP is depleted, which is expected due to α′s participation in a downstream reaction with γ.

To investigate whether using the ATP regeneration machinery with the three-strand system would enable α to react with γ for an extended time, we introduced 1 mM ATP with the regeneration machinery into the reaction solution containing α, β, and γ. We found that the concentrations of γ and β changed over time in a manner following the same qualitative trends we observed when ATP and Rep-X were combined with the same strands. However, the use of ATP regeneration extended the lifetime of the metastable state by approximately 5 h compared to ATP alone (Figure 3c,d). During this 5 h steady state, the rates at which these complexes formed and relaxed back to the ground state were equal, as shown by the plateaus in FAM and Cy3 signal (Figure 3c,d). Also, the use of the ATP regeneration machinery increased the proportion of complexes in the metastable state versus when only ATP was present: about 90% of α:β complexes were unwound, and 75% of free γ was hybridized to α in the presence of the regeneration machinery. This experiment demonstrated that ATP regeneration machinery and Rep-X together can drive a DNA strand displacement reaction network to a steady state in which an output strand is continually freed from a precursor to drive downstream reactions and then recycled.

### Lock Strands Protect Complexes from Unwinding by Rep-X

**Figure 4:**
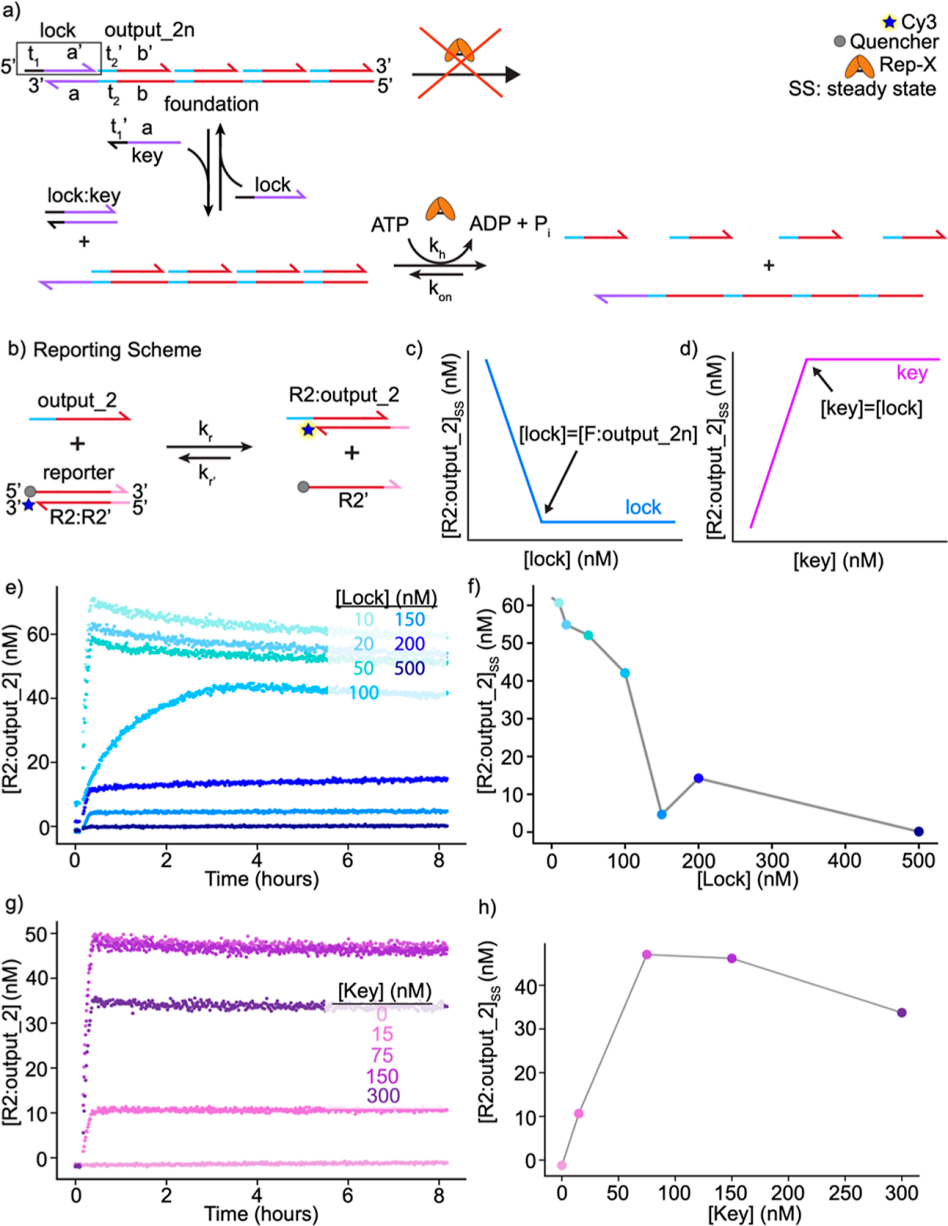
Locking and unlocking a target complex to prohibit or allow Rep-X unwinding. (a) Schematic of the “lock–key” multistrand system. When the lock strand is annealed to foundation:n*output_2, Rep-X is unable to bind to foundation:n*output_2 (where *n* = 1,2,3, or 4 output_2 strands) since the 13-nucleotide 3′ overhang is no longer available. After adding the key, the lock and key strand hybridize, exposing the 3′ overhang of the foundation strand within foundation:n*output_2. Rep-X can bind to and unwind foundation:n*output_2, allowing free output_2 to react with the reporter complex. (b) Reporting scheme for measuring reaction dynamics. Free output_2 from the reaction in (a) can react with the reporter to form R2:output_2, which causes an increase in Cy3 fluorescence. (c) Predicted final R2:output_2 concentration based on the amount of lock annealed to foundation:n*output_2. (d) Schematic of the predicted R2:output_2 concentration based on the amount of key added to a solution containing lock and lock/foundation:n*output_2. (e) Measured concentrations of reacted reporter complex after different concentrations of lock are annealed with foundation:n*output_2. Each sample contained 100 nM Rep-X, 100 nM reporter, 1 mM ATP, and 100 nM foundation:n*output_2; 0, 20, 50, 100, 150, 200, and 500 nM locks were annealed with foundation:n*output_2 in the samples, as labeled. Higher concentrations of lock lead to less output_2 release. The amount of output_2 released plateaus around the 150 nM lock. (f) Average concentrations of R2:output_2 complexes formed after the experiments in (e). In the absence of side reactions, the concentration of R2:output_2 should plateau at 100 nM. (g) Concentrations of reporter complex formed using increasing concentrations of key on “locked” foundation:n*output_2. 100 nM Rep-X, 100 nM reporter, and 1 mM ATP were present in each experiment. 150 nM lock was annealed with 100 nM foundation:n*output_2 for each sample. The samples included 0, 15, 75, 150, and 300 nM key, as labeled. As the concentration of key is increased to 75 or 150 nM, large concentrations of R2:output_2 complexes are produced. A decrease in R2:output_2 is observed once the amount of key exceeds a 1:1 ratio of lock/key. (h) Average concentrations of R2:output_2 complexes formed using different concentrations of key after the experiments in (g). In the absence of side reactions, the concentration of R2:output_2 should plateau at 150 nM. Further details on this nonmonotonic behavior are shown in Figure S4, which includes higher concentrations of key.

The DNA systems studied in Figures 2 and 3 confirm that Rep-X can facilitate dissipative reactions with either ATP alone or ATP coupled with a regeneration reaction, but expanding Rep-X’s implementation to multispecies reactions could pose challenges: Rep-X binds to any 3′ overhang, which with more complicated reactions gives us less control over the system. To see if we could control which strands Rep-X unwinds, we designed a “lock–key” system where a “lock” (a′t_1_) strand is annealed onto the 13-nucleotide 3′ overhang (a) of a long foundation (F) strand which is hybridized with four smaller 13-nucleotide complementary output strands (output_2) (Figure 4a). This “locking” blocks Rep-X from binding to its target complex comprising F-hybridized *n* output_2 strands, where *n* is between 0 and 4 (F:n*output_2; *n* ≤ 4). When the “key” (at_1_’) strand, which is complementary to “lock”, is introduced to the system, it attaches to the 5-nucleotide 5′ overhang of the lock strand and hybridizes to the lock, consequently removing the lock from the target complex. With the 3′ overhang of the F strand now available, Rep-X can bind and unwind the target complex. Upon separation, the free output_2 strands can react with the reporter complex (R2:R2′) (Figure 4b); the output_2 strands can displace R2′ and hybridize with R2. We measured these reaction dynamics by attaching a fluorophore to the 3′ end of R2 and a quencher to the 5′ end of R2’. When output_2 hybridizes with R2, the fluorescence signal spikes, indicating that the “key” has successfully “unlocked” the target complex. The combination of the reporting scheme and F:n*output_2 allows us to observe whether upstream lock-and-key reactions can switch Rep-X-mediated unwinding on and off.

Before testing the entire lock–key system, we tested our reporting scheme. We added increasing amounts of single-stranded output_2 up to 200 nM with 100 nM reporter and found that with more output_2 in solution, more fluorescent R2:output_2 complexes formed (Figure S2). We determined that a max of ∼60% (or 60 nM) of reporter complexes could be unwound by 400 nM of output_2, the amount to be annealed to the foundation strand (Figure S3). After confirming that output_2 successfully displaces the reporter complex, we set out to determine the minimum concentrations of the lock and key strands that respectively suppress or reactivate output_2 release. Determining these concentrations would help us maximize the efficiency of this reaction network and use the smallest amount of lock and key strand possible. We first measured the minimum concentration of “lock” to anneal on F:n*output_2 to achieve 80% repression, i.e., 20% or less of the locked F:n*output_2 is unwound by Rep-X. We observed that as the concentration of lock relative to F:n*output_2 increased, the amount of free output_2 in solution decreased, as expected (Figure 4c). More specifically, we found that we needed at least a 3:2 ratio of lock/F:n*output_2 (in our case, 150 nM of “lock” annealed to 100 nM of F:n*output_2) to achieve ≥80% repression (Figure 4e,f).

### Key Strands Can Reactivate Locked Complexes

Next, we measured how much key strand is required to remove all lock strands from the target complex and produce the same amount of output_2 that is present in the system without lock strands (Figure S3). We expected that increasing the concentration of the key strand that is added to the mixture would increase the concentration of the R2:output_2 complex produced by the reaction up until the concentration of the key equaled the concentration of the lock strand (Figure 4d). We tested the key/lock ratios of 0:1, 0.1:1, 0.5:1, 1:1, and 2:1; in each case, we used 150 nM of lock annealed with 100 nM of the F:n*output_2 complex and 100 nM of reporter. In these experiments, we observed that the concentration of R2:output_2 that formed increased with the key concentration up to a lock/key ratio of 1:1, as predicted. At this ratio, 50 nM of output_2 reacted with the reporter complex to form the fluorescent R2:output_2 complex, compared to 60 nM when no lock or key strands were present (Figure S3). Surprisingly, higher key concentrations caused the concentration of R2:output_2 to decline (Figures 4h and S4b). This decline could be due to an unintended side reaction between output_2 and key (which is detailed in Figure S4c,d) that reduces the amount of output_2 available to react with the reporter complex. Taken together, our results show that double-stranded DNA complexes with 3′ overhangs can be protected from unwinding by Rep-X through hybridization with a lock strand and deprotected by subsequent hybridization with a key strand.

### Thawing Time Affects Rep-X’s Unwinding Activity

During our experiments, we found that maximal unwinding rates by Rep-X could be achieved when its solution was thawed for 60–90 min at room temperature directly from the freezer (−20 °C) before use (Figure S1). This maximal unwinding rate separated around 75 and 70% of the complexes in solution for 60 and 90 min, respectively. While we do not understand why this thawing is beneficial, the protocol for thawing Rep-X was followed for all experiments in this study.

## Conclusions

In this paper, we presented the modified superhelicase, Rep-X, as a new tool for dissipative DNA nanotechnology, specifically to drive networks of strand displacement reactions. Rep-X unwinds strands, which encourages the formation of high-energy transient complexes. These complexes relax to lower-energy configurations after the fuel for Rep-X, ATP, is exhausted. Through studies of three DNA strand displacement systems of increasing complexity, we demonstrated how Rep-X unwinds strands only from complexes with 3′ overhangs and that the single-stranded products of these reactions can then react with other DNA strands. We also showed 3′ overhangs can be protected and deprotected from Rep-X binding by hybridization of a lock strand to the 3′ overhang and that such strands can be removed by a key strand. The enzymes that direct these behaviors, Rep-X and those involved in the ATP regeneration machinery, maintained a steady state for over 5 h, facilitating downstream processes at a range of timescales.

Our results are therefore an important step toward the construction of chemically fueled DNA reaction networks. They also point toward areas along the way that require further investigation. First, while this study elucidated the dynamics of driving to an excited state, a better understanding of the relaxation of complexes to their ground state is needed. In the lock–key system we studied (Figure 4), the reverse reporting reaction, i.e., R2:output_2 returning to R2:R2′, was too slow to characterize the system’s return to the ground state upon ATP depletion. Using a reversible reporting reaction based on the see-saw motif could help solve this issue.^31^ Second, the energy provided by ATP could potentially enable signal amplification; for example, more output_2 strand could be released than the key strand needed to activate the said release. However, in our experiments we did not observe this amplification, which we hypothesize is due to the unintended side reaction between output_2 and the key strand. Third, the integration of enzymes, such as Rep-X into larger DNA reaction networks, will also require further efforts to standardize designs to protect certain complexes from enzymatic activity.

Incorporating Rep-X into more complex DNA reaction networks––although feasible––presents two challenges: first, it requires strategies to protect selected DNA complexes from unwanted unwinding by Rep-X. Since Rep-X selectively targets DNA with 3′ overhangs, the simplest strategy is to design complexes with toeholds on the 5′ end. Replacing DNA nucleotides on the 3′ end with methylated RNA also drastically reduces unwinding by Rep-X.^29^ Second, to develop larger networks, the development of the ODE-based models to predict how the network’s performance is affected by imperfect protection from Rep-X unwinding or unintended side reactions will be important. Because the incorporation of methylated RNA affects the reaction thermodynamics and kinetics, such modeling is not trivial and will require input from quantitative experiments.

## Methods

### DNA Strands

We designed the DNA strands for each system with NUPACK software^32^ under the conditions of 25 °C and 0.5 M Na^+^ and 0.1 M Mg^+^ buffer with 50 or 100 nM DNA. We encoded NUPACK software to match the respective schematics in Figures 2a, 3a, and 4a,b and minimize side reactions (e.g., unwanted hybridizations between strands). After determining the sequences of the strands in each system, we ordered them from Integrated DNA Technologies (IDT) and purchased purified DNA for modified strands with fluorophores or quenchers. We stored all stock solutions from IDT and Rep-X at −20 °C and any diluted DNA solutions at 4 °C.

### Enzyme Synthesis

Rep-X and CPK were synthesized and purified according to the protocol described in refs (28) and (29). In short, recombinant Rep and CPK with 6-histidine His-tags were produced in *E. coli* and purified on a Ni-NTA column. Rep was cross-linked in a bismaleimidoethane solution to obtain Rep-X. The proteins were stored at −80 °C in buffer solution containing 50% glycerol, 600 mM NaCl, 50 mM Tris, pH 7.6.

### Regenerative ATP Synthesis

The regenerative ATP (r-ATP) solution contained 1 mM ATP, 0.2 M phosphocreatine (PCr), 0.1 M 2-mercaptoethanol (βME), 0.2 M creatine phosphokinase (CPK), and 0.1 M MgCl_2_. As Rep-X uses ATP as its fuel, the CPK enzyme catalyzes a reaction that transfers a phosphate from PCr to ADP to form ATP.^28^ This reaction makes creatine a waste product. The constant production of ATP from ADP by CPK and consumption of ATP by Rep-X keep the ATP concentration constant, which Rep-X reuses to continue unwinding DNA.

### Experiments

Experimental results from the three dissipative DNA systems were collected at 25 °C with a sampling frequency of 1 per minute via a Synergy H1 microplate reader using 396 Corning plate wells with a total volume of 25 μL. Each test well contained 1× helicase buffer consisting of 0.1 M Tris HCl, 0.1 M MgCl_2_, and 0.5 M NaCl to preserve the integrity of Rep-X and DNA. We diluted all Rep-X from a 10 to 1 μM stock solution. We thawed Rep-X for 60–90 min in a solution with 1× helicase buffer and water and added it to test wells after collecting initial samples for 5 min on the microplate reader. DNA strands requiring hybridization––R1:output_1 (R1:R1′), foundation:output_2*n, reporter (R2:R2′), α:β, and α:γ––were annealed via PCR before starting any experiments.

### Analysis

After retrieving raw fluorescence data (following the method in 16), we normalized and plotted it using Python. With a known 100 (or 50) nM concentration of DNA in every test well, we used the positive and negative controls to determine the fluorescence of 100 (or 50) nM of the fluorescent DNA strand and the baseline fluorescence (0 nM fluorescent DNA strand). These values were used to normalize the fluorescence readings of samples to determine the DNA concentration (nM). We accomplished this with the following formula in Python
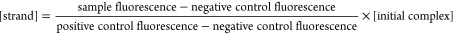


## Supporting Information Available

Experimental details including Rep-X thawing times, lock–key DNA system side reaction, and data containing DNA sequences and concentrations (PDF)

## Abbreviations

dsDNA: double-stranded DNA
r-ATP: ATP regenerative system
Output_1: output strand in double-stranded DNA system (Figure 2)
α: alpha strand in three-strand DNA system
β: beta strand in three-strand DNA system
γ: gamma strand in three-strand DNA system
F: foundation strand in lock–key system
Output_2: output strand in lock–key system (Figure 4)
