# Supplementary material for: Directing Uphill Strand Displacement with an Engineered Superhelicase
Source: ACS Synth Biol. 2023 Oct 16;12(11):3424–32. doi: 10.1021/acssynbio.3c00452 (PMC10661026; doi:10.1021/acssynbio.3c00452)
Supplement: Supplementary file 1 — sb3c00452_si_001.pdf [file sb3c00452_si_001.pdf]

Supplementary Information
Directing Uphill Strand Displacement with an Engineered Super-Helicase

Helena Hall-Thomsen[1], Shavier Small[1], Momcilo Gavrilov[2], Taekjip Ha[2,3,4], Rebecca Schulman[1,5,6,*], Pepijn Gerben Moerman[1,7,*]

[1]Chemical & Biomolecular Engineering, Johns Hopkins University, Baltimore, MD 21218, USA
[2]Biophysics and Biophysical Chemistry, Johns Hopkins University, Baltimore, MD 21218, USA
[3]Biomedical Engineering, Johns Hopkins University, Baltimore, MD 21218, USA
[4]Howard Hughes Medical Institute, Chevy Chase, MD 20815, USA
[5]Computer Science, Johns Hopkins University, Baltimore, MD 21218, USA
[6]Chemistry, Johns Hopkins University, Baltimore 21218, MD, USA
[7]Chemical Engineering and Chemistry, Eindhoven University of Technology, Eindhoven, Netherlands
*Correspondence: rschulm3@jhu.edu, p.g.moerman@tue.nl

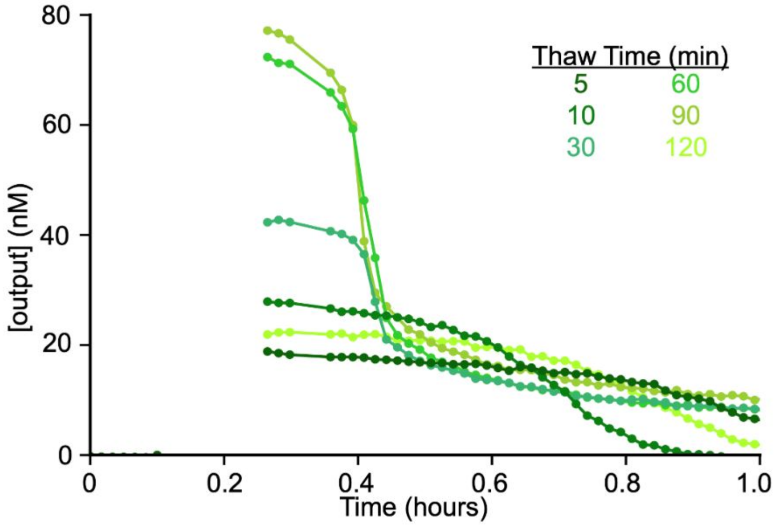

Figure S1. *Amount of output released from initial complex varied over different thawing times of Rep-X.* We thawed Rep-X at room temperature directly from the -20°C freezer for 5, 10, 30, 60, 90, and 120 minutes. Each sample contained 100 nM of R1:output_1, 100 nM Rep-X, and 1 mM ATP. We observed the highest Rep-X unwinding rate or efficiency at 90 minutes with an unwound fraction of ~75%. From these results, we decided to follow a 60 to 90 minute room temperature thawing Rep-X protocol for each of the three DNA systems.

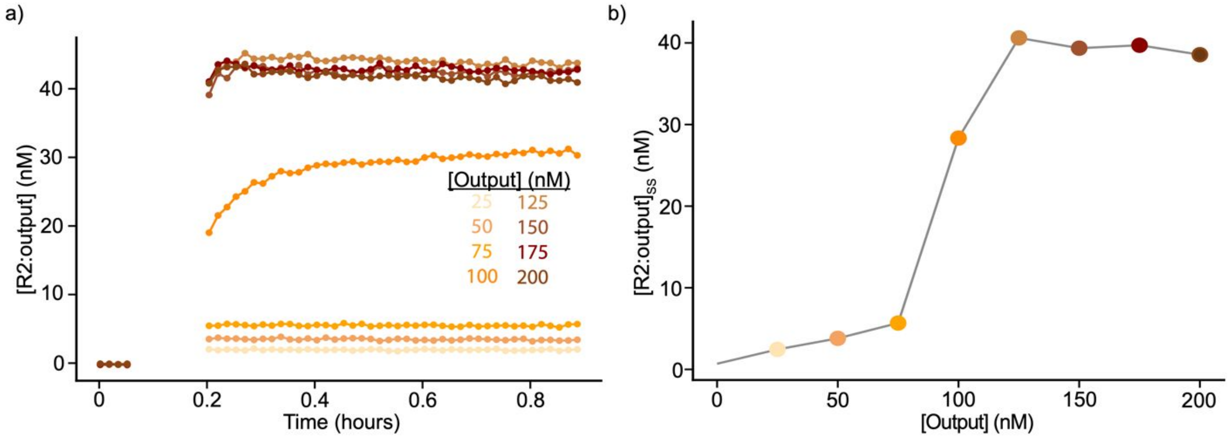

Figure S2. a) *Proportion of R2:output_2 complexes formed using different concentrations of output_2.* With 100 nM of reporter,1 mM of ATP and varying free, single-stranded output_2 concentrations of 25, 50, 75, 100, 125, 150, 175, and 200 nM, the amount of reporter displaced by output_2 plateaus at ~45% with 125 nM of output_2. **b**) *Average amount of R2:output_2 complexes formed with varying concentrations of output.*

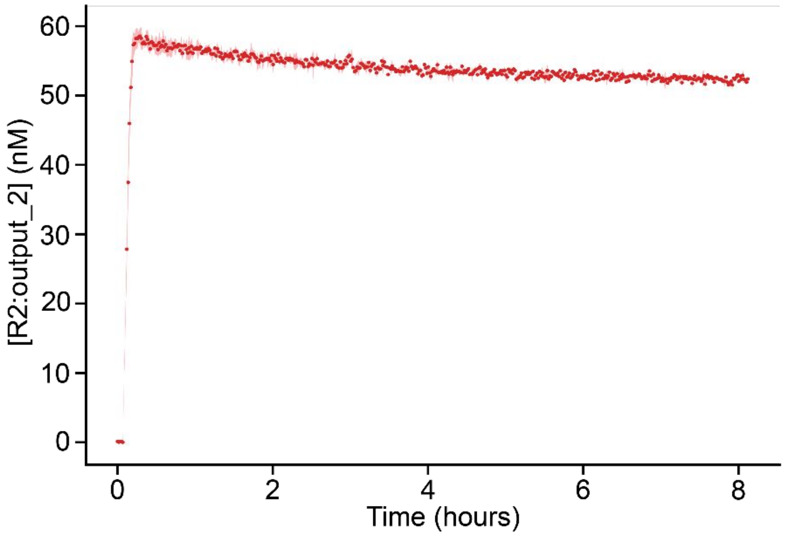

Figure S3. *Evaluating the effectiveness of the reporting scheme as measure of "lock-key" reaction dynamics.* With 400 nM of free, single-stranded output_2 and 100 nM of reporter, ~60% of available reporter was unwound by the hybridization of output_2 to R2. Red shading represents standard deviation of the duplicate samples, however, there is not enough deviation between the duplicates to be easily visible.

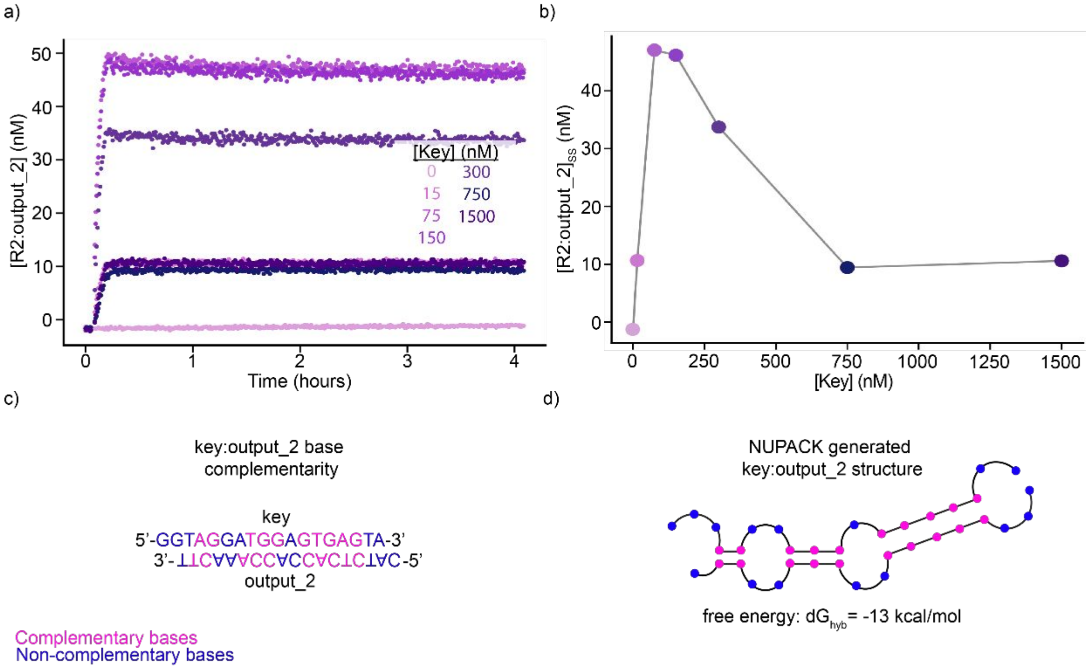

Figure S4. **a**) *Concentrations of reporter complex formed using increasing concentrations of key on "locked" foundation:output_2n.* 100 nM Rep-X, 100 nM of reporter, and 1 mM ATP were present in each experiment. 150 nM of lock was annealed with 100 nM foundation:output_2n for each sample. The samples included 0, 15, 75, 150, 300, 750, and 1500 nM key as labeled. As the concentration of key is increased to 75 or 150 nM, large concentrations of R2:output_2 complexes are produced. A decrease in R2:output_2 is observed once the amount of key exceeds a 1:1 ratio of lock:key. **b**) *Average concentrations of R2:output_2 complexes formed using different concentrations of key after the experiments in (a).* **c**) *Complementarity of key:output_2 complex.* There are 10 complementary bases (pink) between output_2 and key. The formation of key:output_2 complexes prevents R2:output_2 complexes from forming, leading to a decrease in fluorescence since less output_2 reacts with R2. **d**) *NUPACK generated structure of key:output_2*. Simulated in a solution of 300 nM key and 400 nM output_2, key:output_2 complexes form ~60% of the ensemble. The free energies were calculated using a reference concentration of 55.6M (water in water) since we use an aqueous solution at low concentrations of solute.

Table S1. DNA strand sequences.

Strand Name	Sequence	# of nt	Purification
Output_1	\5FAM\TGAAGTTTGGTGGTG AGATG	20	Standard desalting
R1’	CACCACCAAACTTCA\3IowBk\	15	HPLC (High Performance Liquid Chromatography)
Q1	/56FAM/TGAAGTTTGGTGGTGAGATG	20	Standard desalting
Q2	CACCACCAAACTTCA/3IABkFQ/	15	HPLC
Alpha (α)	GTGTAAGTA GGA GTGAGGTGAGG\3IowBk\	23	HPLC
Q3	/5IABkFQ/CACCACCAAACTTCA	15	HPLC
Beta (β)	\5FAM\CCTCACCTCACT CCT ACTTACAC TTTTTTTTTT	33	Standard desalting
Gamma (γ)	\5Cy3\CCTCACCTCACTACTTACAC	20	HPLC
Foundation (F)	AAGTTTGGTGGTG AGATG AAGTTTGGTGGTG AGATG AAGTTTGGTGGTG AGATG AAGTTTGGTGGTG AGATGGGTAGGATGGAGT	85	Standard desalting
Output_2	CATCTCACCACCAAACTT	18	Standard desalting
Lock	TACTC ACTCCATCCTACC	18	Standard desalting
Key	GGTAGGATGGAGT GAGTA	18	Standard desalting
R2	/5cy3/TGAAGTTTGGTGGTG /i2MOErA//i2MOErG//i2MOErA//i2MOErT//i2MOErG/	15	HPLC
R2’	CACCACCAAACTTCA/3IABkFQ/	15	HPLC

[1]Purified strands have attached fluorophores or quenchers.
[2]Complementary strands are denoted by an apostrophe for reporter complexes or by matching colors.
[3]All strands are listed from 5’ to 3’ direction.

Table S2. Experimental details of SI Figure 1.

Sample (Concentration)	Amount Added (μL)							
	Positive Control	Negative Control	Test Wells (Varied Rep-X thaw times in mins)					
			5	10	30	60	90	120
R1:output_1 (1 μM)		2.5	2.5	2.5	2.5	2.5	2.5	2.5
Q1 (1 μM)	2.5							
Q2 (1 μM)	2.5							
Rep-X (1 μM)			2.5	2.5	2.5	2.5	2.5	2.5
ATP (10 mM)	2.5	2.5	2.5	2.5	2.5	2.5	2.5	2.5
Helicase Buffer (*10x*)	2.5	2.5	2.5	2.5	2.5	2.5	2.5	2.5
Water	15	17.5	15	15	15	15	15	15

Table S3. Experimental details of Figure 1c.

Sample (Concentration)	Amount Added (μL)						
	Positive Control	Negative Control	Test Wells (Varied ATP Concentration)				
			0.1 mM	0.2 mM	1 mM	2 mM	5 mM
R1:output_1 (1 μM)		2.5	2.5	2.5	2.5	2.5	2.5
Q1 (1 μM)	2.5						
Q2 (1 μM)	2.5						
Rep-X (1 μM)			2.5	2.5	2.5	2.5	2.5
ATP (5 mM)			0.5	1			
ATP (10 mM)	2.5	2.5			2.5	5	
ATP (50 mM)							2.5
Helicase Buffer (*10x*)	2.5	2.5	2.5	2.5	2.5	2.5	2.5
Water	15	17.5	17	16.5	15	12.5	15

Table S4. Experimental details of Figure 1d.

Sample (Concentration)	Amount Added (μL)			
	Controls		Test Wells (1 mM ATP)	
	Positive	Negative	No regenerative system	With regenerative system
R1:output_1 (1 μM)		2.5	2.5	2.5
Q1 (1 μM)	2.5			
Q2 (1 μM)	2.5			
Rep-X (1 μM)			2.5	2.5
ATP (10 mM)	2.5	2.5	2.5	
ATP with regeneration machinery (10 mM)				2.5
Helicase Buffer (*10x*)	2.5	2.5	2.5	2.5
Water	15	17.5	15	15

Table S5. Experimental details of Figure 2c, 2d.

Sample (Concentration)	Amount Added (μL)					
	5-FAM		5-Cy3		Test Wells (1 mM ATP)	
	Positive Control	Negative Control	Positive Control	Negative Control	No regenerative system	With regenerative system
α (1 μM)						
β (1 μM)	2.5					
γ (1 μM)			2.5			
Q3 (1 μM)	2.5		2.5			
α:β (1 μM)		2.5				
α:γ (1 μM)				2.5		
α:β + γ (1 μM)					2.5	2.5
Rep-X (1 μM)					2.5	2.5
ATP (10 mM)	2.5	2.5	2.5	2.5	2.5	
ATP with regeneration machinery (10 mM)						2.5
Helicase Buffer (*10x*)	2.5	2.5	2.5	2.5	2.5	2.5
Water	15	17.5	15	17.5	15	15

Table S6. Experimental details of SI Figure 2.

Sample (Concentration)	Amount Added (μL)		
	Controls		Test Wells (Duplicated)
	Positive	Negative	
R2:R2' (1 μM)	2.5	2.5	2.5
F:output_2n (1 μM)		2.5	2.5
Output_2 (1 μM)	10		
Rep-X (1 μM)			2.5
ATP (10 mM)	2.5	2.5	2.5
Helicase Buffer (*10x*)	2.5	2.5	2.5
Water	7.5	15	12.5

Table S7. Experimental details of SI Figure 3a, 3b.

Sample (Concentration)	Amount Added (μL)									
	Controls		Test Wells (Varied Output Concentration in nM)							
	Positive	Negative	25	50	75	100	125	150	175	200
R2:R2' (1 μM)		2.5	2.5	2.5	2.5	2.5	2.5	2.5	2.5	2.5
R2 (1 μM)	2.5									
F:output_2n (1 μM)										
Output_2 (500 nM)			1.3	2.5	3.8					
Output_2 (1 μM)						2.5	3.1	3.8	4.4	5
Rep-X (1 μM)										
Helicase Buffer (*10x*)	2.5	2.5	2.5	2.5	2.5	2.5	2.5	2.5	2.5	2.5
Water	10	20	18.8	17.5	16.3	17.5	16.9	16.3	15.6	15

*Some numbers rounded.

Table S8. Experimental details of Figure 3e, 3f.

Sample (Concentration)	Amount Added (μL)							
	Test Wells (Varied Lock Concentration)							
	none	10 nM	20 nM	50 nM	100 nM	150 nM	200 nM	500 nM
R2:R2' (1 μM)	2.5	2.5	2.5	2.5	2.5	2.5	2.5	2.5
F:output_2n (1 μM)	2.5							
Lock-10:F:output_2n (1 μM)		2.5						
Lock-20:F:output_2n (1 μM)			2.5					
Lock-50:F:output_2n (1 μM)				2.5				
Lock-100:F:output_2n (1 μM)					2.5			
Lock-150:F:output_2n (1 μM)						2.5		
Lock-200:F:output_2n (1 μM)							2.5	
Lock-500:F:output_2n (1 μM)								2.5
Rep-X (1 μM)	2.5	2.5	2.5	2.5	2.5	2.5	2.5	2.5
ATP (10 mM)	2.5	2.5	2.5	2.5	2.5	2.5	2.5	2.5
Helicase Buffer (10x)	2.5	2.5	2.5	2.5	2.5	2.5	2.5	2.5
Water	12.5	12.5	12.5	12.5	12.5	12.5	12.5	12.5

*Same controls as Table 5.
*Lock-10:F:output_2 indicates 100 nM of lock was annealed to the F:output_2n complex.

Table S9. Experimental details of Figure 3g, 3h and SI Figure 3c, 3d.

Sample (Concentration)	Amount Added (μL)						
	Test Wells (Varied Key Concentration)						
	none	15 nM	75 nM	150 nM	300 nM	750 nM	1.5 μM
R2:R2’ (1 μM)		2.5	2.5	2.5	2.5	2.5	2.5
Lock-150:F:output_2n (1 μM)	2.5	2.5	2.5	2.5	2.5	2.5	2.5
Key (1 μM)		0.4	1.9	3.8	7.5		
Key (10 μM)						1.9	3.8
Rep-X (1 μM)	2.5	2.5	2.5	2.5	2.5	2.5	2.5
ATP (10 mM)	2.5	2.5	2.5	2.5	2.5	2.5	2.5
Helicase Buffer (10x)	2.5	2.5	2.5	2.5	2.5	2.5	2.5
Water	12.5	12.1	10.6	8.8	5	10.6	8.8

*Same controls as Table 5.
*Some numbers rounded.
